# Chronic pain self-management in middle-aged and older adults: A collective intelligence approach to identifying barriers and user needs in eHealth interventions

**DOI:** 10.1177/20552076221105484

**Published:** 2022-06-07

**Authors:** Paul M O’Reilly, Owen M Harney, Michael J Hogan, Caroline Mitchell, Brian E McGuire, Brian Slattery

**Affiliations:** 1 School of Psychology, 8799 National University of Ireland, Galway, Ireland; 2 Centre for Pain Research, 8799 National University of Ireland, Galway, Ireland; 3 Division of Pain Medicine, Galway University Hospital, Galway, Ireland; 4 School of Psychology, 8818 Dublin City University, Dublin, Ireland

**Keywords:** Collective intelligence, eHealth, digital health, older adults, chronic pain

## Abstract

**Objectives:**

eHealth refers to health services and health information delivered or enhanced through the internet and related technologies. The number of eHealth interventions for chronic pain self-management is increasing. However, little evidence has been found for the overall efficacy of these interventions for older adults. The aim of the current study was to use a Collective Intelligence approach to identify the barriers and specific user needs of middle-aged and older adults using eHealth for chronic pain self-management.

**Methods:**

A Collective Intelligence workshop was conducted with middle-aged and older adults to generate, clarify, select, and structure ideas in relation to barriers to eHealth use and specific design requirements for the purposes of chronic pain self-management. Prior to attending the workshop, participants received a trigger question requesting the identification of five barriers to eHealth use for chronic pain self-management. These barriers were categorised and presented to the group along with barrier-related scenarios and user need prompts, resulting in the generation of a set of ranked barriers and a set of user needs.

**Results:**

A total of 78 barriers were identified, from which six categories emerged: Content, Support, Technological, Personal, Computer Literacy and Accessibility. Additional idea-writing and group reflection in response to these barriers revealed 97 user needs.

**Conclusion:**

This is the first study to use Collective Intelligence methods to investigate barriers to eHealth technology use and the specific user needs of middle-aged and older adults in the context of chronic pain self-management. The results of the current study provide a platform for the design and development of enhanced eHealth interventions for this population.

## Introduction

eHealth or digital health is defined as health-related information and services delivered or enhanced through the internet and related technologies (e.g. laptop computers and smartphones).^1,2^ More specifically, eHealth interventions are remotely delivered health treatments designed to increase health behaviours and improve disease management. The benefits of digital health interventions are evident with research highlighting their ability to provide flexible, effective, cost saving, scalable and safe interventions to improve health and healthcare.^3–5^ For example, they have been successfully shown to enable users to become better informed about their own health, change users perceptions and cognitions regarding health and healthcare, allow users to assess and monitor specific health states or health behaviours, and improve user social support through improving communication with healthcare professionals and the sharing of experiences with others in a similar situation.^4^ For these reasons, eHealth interventions are being used with increasing frequency across a variety of health conditions and behaviours, including HIV prevention,^6^ weight management,^7^ medication adherence^8^ and chronic pain.^5,9–11^

### Chronic pain

Chronic pain (CP) is defined as ‘pain that persists or recurs for more than 3 months’.^12^ People who live with CP often live with long-term disability, particularly experiencing interference to their everyday activities, and many develop psychological difficulties, such as depression and anxiety.^13^ Self-management, the provision of education and supportive interventions by healthcare professionals to increase patients’ skill and confidence in managing their health problems^14^ has been effective in improving patient health outcomes for people with CP.^5,15^

Recently, research has shown that eHealth technology can be efficacious in improving CP self-management,^5^ for example, maintaining treatment gains during multidisciplinary treatment for chronic back pain^9^; changing health-related behaviours and improving health status measures for arthritis.^16^ Specifically, interventions delivered via these technologies have shown the ability to reduce CP intensity,^17^ as well as pain-related disability^18^ and pain-related interference.^19^ Although eHealth interventions for the self-management of CP have been implemented across all age groups, a large number of these interventions have favoured younger populations,^20,21^ or have included older people but have not analysed their outcomes separately,^22^ with only a small number of interventions carried out with older adults.^23^ Research has recommended further evaluation of digital health interventions with older people with CP.^24^

### eHealth technology design for older adults with CP

The global population will soon be in a situation where adults over 65 years of age will outnumber children under the age of 5.^25^ As people get older, they often live with painful conditions such as osteoporosis, arthritis and back pain, which can lead to increased levels of disability, decreased levels of mobility and impaired quality of life.^26^ While some success has been found with eHealth technology interventions to assist older adults to self-manage their CP,^27,28^ there is little evidence for the overall efficacy of these types of interventions for this population.^23^ With the success that eHealth technology has shown across younger CP populations,^20,21^ it is curious why similar success not been found with older populations. Indeed, given the points made regarding global population ageing, increasing levels of long-term illness and the efficacy of eHealth interventions more generally, it could be argued that the successful adoption of eHealth technology by older populations with CP is an important next step in the progression of healthcare.

One reason proposed for the unsuccessful adoption of eHealth technology by older adults is the way in which eHealth technologies are designed.^29^ While user involvement in the general design of medical devices and healthcare technology can lead to increased usability and quality,^30,31^ the design processes of these technologies tend to give precedence to the voices and opinions of the designers.^32^ As a consequence, healthcare technology design and development processes often lack the consideration of the preferences of older adults and the consideration in general of the compatibility of the technology with the people for whom it is intended.^33^

Research has also shown that these technologies are often designed according to a hegemonic idea of age, with age diversity not taken into account and older adults seen as an ‘other’ group who are not considered as a distinct cohort for consideration in the design process.^32^ A recent systematic review, examining smartphone applications designed to support pain self-management for older adults found that despite the availability of a large number of mobile-apps, few offered older adult-specific usability (e.g. functions enabling the enlarging of app screen size or font were not provided in any of the apps) and that in general the older adult-specific usability of pain self-management apps available could be classified as moderate at best.^29^ The researchers, therefore, recommended that future work in the area of pain self-management be considerate of the usability needs of older adults in future pain app development endeavours.^29^

### Collective intelligence methodology for technology design

In the context of multidisciplinary research, one methodological approach that has garnered support as a framework for extrapolating consensus from groups is Collective Intelligence (CI).^34^ CI refers to knowledge that emerges from a group’s combined capability and efforts to understand and address a shared problem, through the facilitated implementation of a specific range of methodologies.^35^ The CI approach helps to support high-quality interdisciplinary work as it carefully delineates content and process roles, assigning to content experts (i.e. workshop participants) the responsibility for contributing ideas, and to the CI facilitation team responsibility for choosing and implementing selected methodologies for generating, clarifying, structuring, interpreting, and developing ideas. The CI methodologies were designed as a facilitation system to assist groups in dealing with complex situations and aid groups in the developing of outcomes that integrate contributions from individuals with diverse points of view, perspectives and backgrounds.^36^ Examples of such CI methodologies are idea writing, nominal group technique, field representations and interpretive structural modelling.^37^ Importantly, these CI methods may be paired with methods such as Scenario-Based Design (SBD)^38^ and user stories^39^ in design contexts. When combined, these methods help a group to first understand the problematic components of a system (e.g. barriers to the use of CP self-management interventions), and build upon that understanding to move into design work aimed at developing and refining operational specifications (e.g. specific design needs for CP self-management interventions). CI has been applied in multiple user needs and participatory design contexts, including developing a national well-being measurement framework,^40^ understanding and overcoming barriers to the design of personalised nutrition products and services for older adults,^41^ and understanding and overcoming barriers to the design of open data platforms for citizens and public administrators.^42^

The current eHealth literature highlights limited evidence for the efficacy of eHealth interventions for CP self-management among older adults,^23^ the lack of consideration in the design process for the intended user^32,33^ and the need to consider the usability needs of older adults in future eHealth pain app development.^29^

The current study employed the CI methodology to engage with end users to identify, clarify, and collate specific barriers, and associated user needs, with respect to the uptake of eHealth interventions to assist middle-aged and older adults with the self-management of their CP. As such, one of the primary advantages of using CI in this context was the facilitation of communication between various potential CP self-management tool users in relation to usage possibilities and the challenges that may arise for different stakeholders. By using CI to identify barriers to accessing, understanding, and using eHealth technology for the self-management of CP, the current research identifies and organises specific needs of middle-aged and older adult users, so that future eHealth technologies can be designed with these user needs in mind, making them more useable, rewarding and effective for middle-aged and older adults with CP.

## Methods

### Ethics

Prior to data collection, ethical approval was granted by the Ethics Committee of the School of Psychology, NUI Galway, and all participants provided written informed consent.

### Participants

A convenience sample of 17 participants (12 female and five male) were recruited through the Pain Clinic in University Hospital Galway and the Centre for Pain Research (CPR), NUI Galway. The inclusion criteria required that participants were 50 years and above and suffer from CP. The cut-off of 50 years of age for participant recruitment was based on similar research in the area of middle-aged and older adults’ and technology use.^43,44^ The exclusion criteria were participants with a life-limiting condition, severe mental health problems, or cognitive or language difficulties that would prevent participants from giving informed consent. Participants recruited from the Pain Clinic were identified according to the inclusion and exclusion criteria by the Registered Advance Nurse Practitioner and were recruited via telephone. Participants recruited through the CPR were recruited from a previous eHealth research study via email and telephone. All participants were representative of the CP population in the region.

### Design

A workshop was conducted using a combination of CI and SBD approaches as well as user stories,^39^ to gather and integrate the views and perspectives of middle-aged and older adults (*n* = 17) in relation to eHealth use for the purposes of CP self-management. CI is a facilitated thought and action mapping process that helps groups to develop outcomes that integrate contributions from individuals with diverse views, backgrounds and perspectives.^45,46^ The CI methodology is comprised of a number of methods and tools, which are selected and employed by the facilitation team based on the specific goals of the CI design project. In the current study, *ideawriting* and *field representation* methods were employed, in conjunction with SBD and user stories.

Ideawriting^37^ is a method in which small groups (typically 4–6 persons), formed by dividing a larger group into several working teams, work together to develop ideas and subsequently explore the nature of those ideas through open discussion. Ideawriting is a five steps process: (1) presentation of a stimulus question to experts; (2) silent generation of ideas in writing by each participant working alone; (3) exchange of written sheets of ideas among all group members, with opportunity for individuals to add ideas as they read others’ papers; (4) discussion and clarification of unique ideas; and (5) an oral report of the ideas generated by each working group.

Field representations were generated in advance of the CI workshop using the *paired comparison method*^45,47^ to compare barriers in pairs and identify categories of related barriers. The *paired comparison method* is a process of systematically comparing pairs of barrier statements, and assessing each pair for conceptual similarity. Through this exhaustive process, categories of conceptually similar barriers to merger, which are then labelled with category headings.

SBD is a group of techniques in which the future use of a system is concretely described at an early point in its development process through narrative descriptions of envisioned usage scenarios.^38^ These flexible and generative scenarios are designed to elicit constructive cognitive processes and the development of creative and bespoke solutions that are suitable for the specific context, and thus do not specify fixed solutions. SBD is an iterative approach to interactive systems design and analysis. The process encourages explicit reasoning about how users interact with technology and prompts the exploration of specific requirements and affordances which users will need in various types of interactions. SBD also may be used to facilitate consideration of design trade-offs throughout development, including trade-offs between the potential impact of design decisions and the feasibility of the design options.^38^

Following reflection by participants on such scenarios, the template for future solutions may take the form of user stories. User stories have been proposed as a way to enhance communication between the stakeholders and the developers.^39^ These user stories, which involve generating specific needs for specific user types, allow designers to know more about who the user is and what problems they are seeking to address, and thus can inform the design of better systems.^48^

The outlined approach, beginning with a CI exploration of barriers, before an SBD-user stories generation of user needs, is specifically aimed at avoiding solution-first problem-solving. Solution-first approaches are often problematic, as designers may develop solutions prematurely, without careful analysis of the challenges and needs of the targeted cohort of users.^49^ By beginning with a CI analysis of barriers, a rich context was provided in which to ground the subsequent identification of user needs, and thus the potential for future design and development.

### Materials

A series of standardised measures typically used in pain research (The Multimorbidity Illness Perceptions Scale,^50^ 2013, The Medical Outcomes Short Form-12 (SF-12),^51^ The CP Grade Questionnaire,^52^ The PHQ-9 depression scale^53^ and The GAD-7 anxiety scale^54^) were administered to ensure that participants were somewhat representative of the CP population. Participants also provided standard demographic information such as sex and age, were asked to identify (from a checklist) how they rated their general health, where they experienced pain and any pain-related conditions they had (such as arthritis), as well as whether they utilised a range of services in the previous 12 months (e.g. GP, physiotherapy). Participants were also asked about their level of healthcare technology use in the last 12 months, the type of healthcare technology that they are most familiar with and if they believed that these technologies can help them with their illness.

## Procedure

### Stage 1 – Pre-CI workshop idea generation

Two weeks in advance of the CI workshop, participants were issued an email containing the trigger question ‘What are the barriers to accessing, understanding and using online or internet-connected technology that assist older adults self-manage and live well with their chronic pain?’. Participants were asked to generate five barriers in response to this question and return their answers via email. Upon receipt of these, barriers POR and OH employed the Paired Comparison Method^45^ to systematically generate categories of conceptually similar barriers. These categories were subsequently reviewed and agreed upon by POR, OH and BS. With the trigger question, participants were also issued a questionnaire which asked for basic information about themselves, details of their CP, details about how they were coping with their CP and information about the types of eHealth technology that they have used. Participants were asked to fill this out and bring it to the workshop.

### Stage 2 – Review of barriers

On the morning of the CI workshop, participants were welcomed into a room in the School of Psychology, NUI Galway and divided into groups of four or five. During the first phase of the workshop, participants were presented with handouts containing the barrier categories and asked to discuss within their groups what they felt the most substantial barriers to using eHealth technology were. This process began with silent, individual reflection, allowing each participant to review the full set of barriers before the discussion began. The facilitation team circulated the room during discussions, offering guidance and instruction where necessary. Following this period of reflection and discussion, each group nominated a spokesperson to present to the room an overview of what their group had discussed, including elaborations on barriers that the group agreed to be most impactful. Participants were also individually asked to identify what they felt were the two most significant barriers in each category by placing an ‘X’ beside those barriers on the handout.

### Stage 3 – Generation and prioritisation of user needs

**Figure 1.**
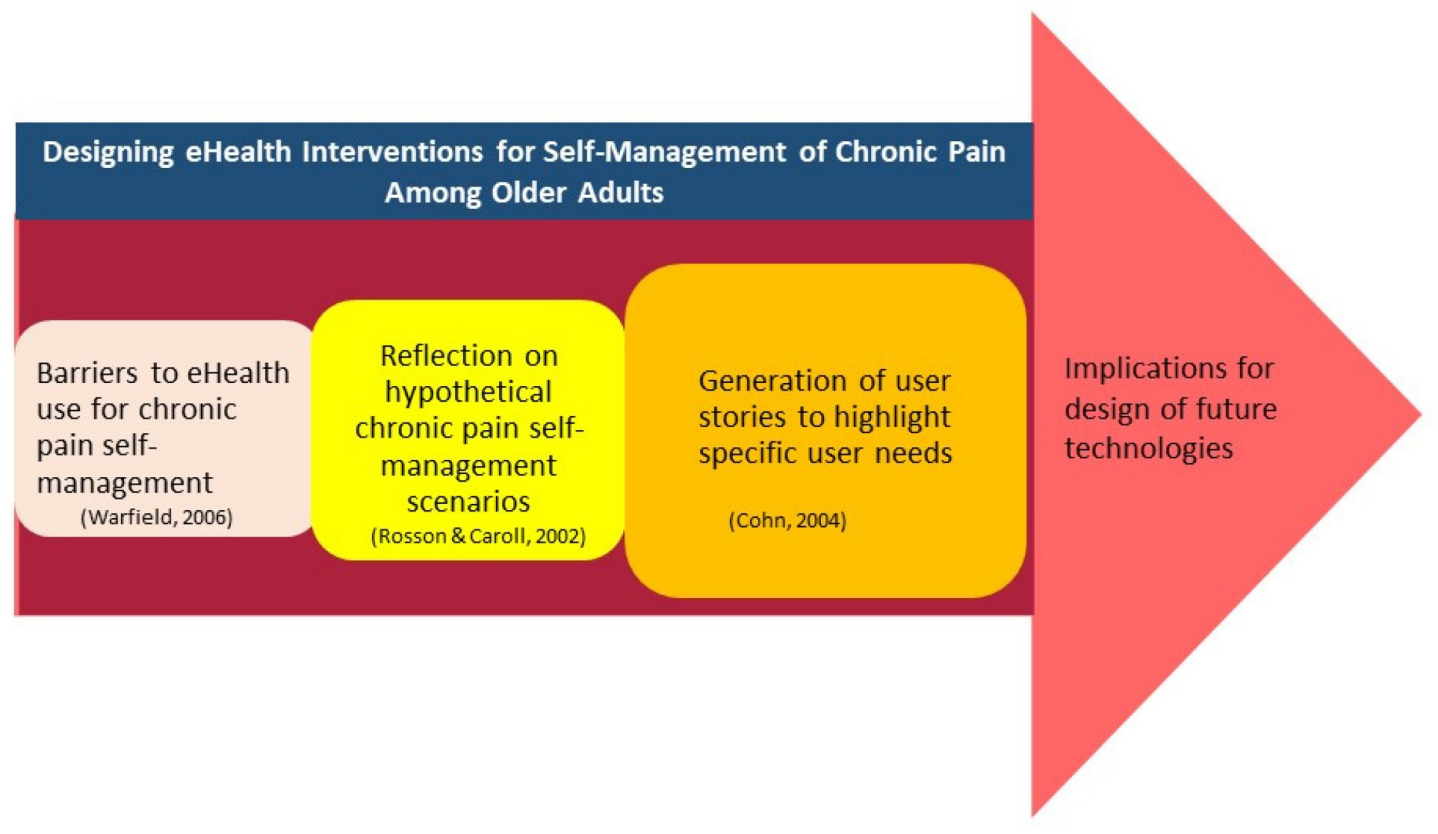
Phases in the collective intelligence scenario-based design process.

Following the review and prioritisation stage of the workshop, participants engaged in a facilitated SBD and user stories exercise. In advance of the CI session, the research team and facilitation team worked together to design a set of four scenarios to frame idea generation around user needs in the context of eHealth CP self-management interventions. These scenarios (see Appendix B) were designed in line with guidelines^38^ to be concrete, flexible and generative, and did not specify fixed solutions. In SBD, scenarios are used to elicit constructive cognitive processes and the development of creative and bespoke solutions in specific design contexts, as presented in the scenario. In the current CI application, the SBD process provided an opportunity for collaborative analysis and elaboration of eHealth user needs in the exchange between workshop participants. The scenarios, including challenges faced by multiple actors (general practitioners, pain specialists, patients) were used to prompt thinking in relation to user needs from a variety of perspectives and different circumstances. Participants were asked to generate their user needs, on handouts provided, in the following format: ‘As a user_______, I want ______so that I can________’ (see Appendix D). The *ideawriting* method was employed by the facilitator during this phase, to guide participants through the process of generating user needs. Following a period of initial silent idea generation, participants were facilitated in discussing these user needs within their groups. Each group was asked to the first review, clarify, and elaborate upon the user needs generated within their group, before working to arrive at a subset of needs that the group collectively believed to be most important and have the highest potential impact. Once again, each group nominated a spokesperson to present an overview of the group discussions, and key identified user needs, to the room. The user needs to be chosen by each group were written on A5 post-it notes and displayed on the walls around the room. Figure 1 presents a flow diagram of the procedure.

### Data analysis

Workshop materials were collated by the research team (POR, OH and BS). Additional barriers that were identified during the workshop were categorised according to the pre-existing barrier categories and added to those relevant categories. Using the barrier sheets that were collected from participants at the end of the workshop, the top three barriers that were highlighted as the most important barriers in each category were identified. The User Needs to be identified by participants were collated from the User Needs sheets and A5 post-it notes and categorised first according to their relevance to the barrier categories and then to their relevance to the identified top three barriers in each category. The audio recordings of the workshop were transcribed verbatim by the research team and quotes relevant to the top three barriers and relevant user needs were extracted.

## Results

A total of 17 middle-aged and older adults (12 female and five male) agreed to participate and gave informed consent in the current study. Based on the returned descriptive data of 13 participants, the average age of participants was 61.3 years (*SD* *=* 7.69), ranging from 50 years to 76 years. When asked about their healthcare technology use in the previous 12 months, 38% of participants reported accessing healthcare technology via medical devices (e.g. home blood pressure monitor), 31% via smartphones (e.g. digital diary for keeping track of pain severity, pedometer) and 38% via internet-based healthcare program (e.g. online pain management course). These types of healthcare technology were accessed rarely by 54%, infrequently by 39% and frequently by 23%. Participants mostly used portable computers (62%) and smartphones (38%) to access healthcare technology, and 54% believed that healthcare technologies could help them with their illnesses. See Table 1 for participants’ descriptive information.

**Table 1.** Participants’ descriptive information.

*n* = 13				
Measure	Percent	Mean	(SD)	Range
Age		61.2	7.34	26
Gender				
Female	71			
Male	29			
General health				
Poor	15			
Fair	31			
Good	31			
Very good	8			
Excellent	8			
Conditions				
Back pain	92			
Neck pain	77			
Other pain	69			
Osteoarthritis	31			
Rheumatoid arthritis	8			
Other musculoskeletal	38			
Sleep disorder	46			
Depression	38			
Anxiety	38			
Multiples				
Treatment burden		14.6	6.61	19
Prioritising conditions		15.5	3.21	9
Causal links		11.9	2.88	10
Activity limitations		11.1	3.67	11
Emotional representations		22.6	2.88	21
Summary scale		66.7	27.4	63
SF-12				
Mental component		45.3	8.46	26.7
Physical component		35.5	6.34	23.14
Chronic pain grade				
Pain intensity		70.8	17.1	50
Disability score		64.8	26.5	77
Disability points		3.77	2.28	6
PHQ-9		12.4	5.82	12
GAD-7		6.15	4.81	15
Healthcare usage				
General practitioner	100			
Physiotherapy services	69			
Pain clinic	62			
Psychological services	54			
Practice nurse	31			
Outpatient clinic	31			
Inpatient day admission	31			
Inpatient overnight stay	31			
Technology usage				
Medical device	37			
Internet-based program	38			
Smartphone	31			
Device usage				
Portable computer	62			
Smartphone	38			
Personal computer	8			

### Collective intelligence

**Figure 2.**
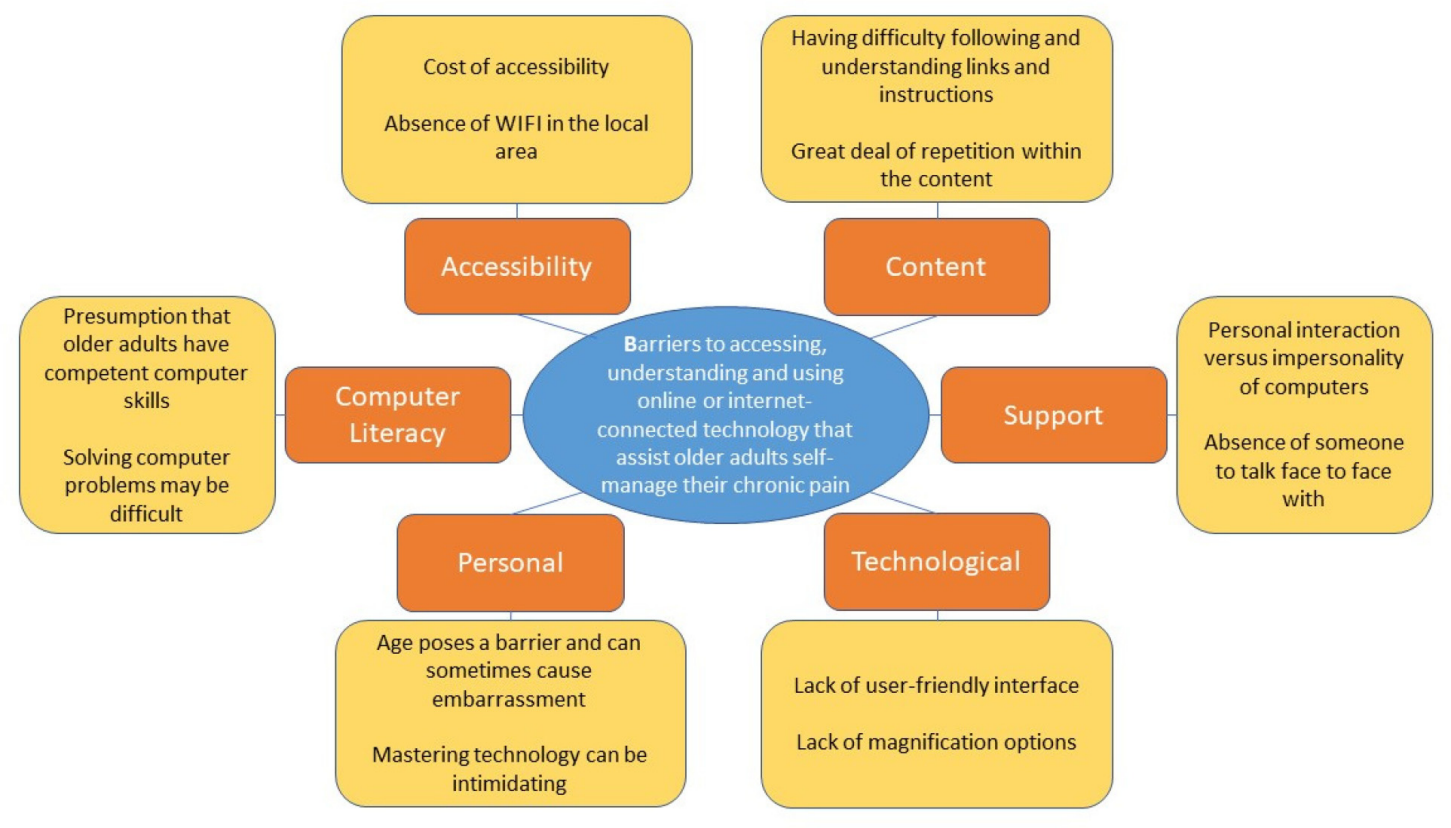
Categories of barriers.

In total, 78 barriers to the use of eHealth technology for the purpose of CP self-management for older adults emerged across six barrier categories: Content, Support, Technological, Personal, Computer Literacy and accessibility (see Appendix A, Tables 1 to 6). Figure 2 presents a sample of barriers within each category.

#### Content barriers

In relation to *Content*, participants noted that there can be difficulty following and understanding the instructions provided within eHealth programs and that there is often a lack of guidelines or information to help people to overcome difficulties that they may encounter. Participants noted that the timeline allocated for eHealth programs is often insufficient for the successful completion of these programs and that there can be repetition and a lack of flow and logic to the program content, all of which can act as barriers. The lack of information relating to the content of eHealth programs was also identified as a barrier, as was the uncertainty as to whether the program content would be relevant to participants and their condition. The inability to receive individual-specific feedback and give feedback to the program coordinator in relation to the effectiveness of the program was also identified as a barrier by participants. Participants noted that eHealth programs for CP self-management often neglect important aspects of their condition that can hugely impact their lives such as CP-related depression and the difficulties that so many people suffering from CP have with family and friends misunderstanding the seriousness of their condition. Participants also felt that their needs as middle-aged and older adults were not being met, specifically that eHealth programs did not offer information to help them cope with their CP on a daily basis.

The three barriers that participants felt were the most important *Content* barriers were:

1. Absence of acknowledgement that counselling in primary care can be of some benefit to CP patients (seven votes).
2. Failure to acknowledge that depression can be part of CP (five votes).
3. Inadequate information to assist older adults self-manage their CP (three votes).
  Multiple choice answers can often be too similar (three votes).
  Lack of information about possible new treatments, therapies and medication for CP (three votes).

#### Support barriers

In relation to the *Support* category, participants identified the lack of human contact that can occur when using eHealth programs and the general impersonality of the use of computers as barriers to the use of eHealth programs. Specifically, participants identified the inability to contact someone for advice or clarification if they felt confused by or misunderstood some aspect of the program as well as the lack of face-to-face contact with someone. Participants also identified feelings of abandonment once the program had finished and the frustration of carrying out the learned elements of the program on their own afterwards as barriers to eHealth use. Some participants also noted the lack of empathy provided by eHealth technology use, which left some feeling like a statistic.

The three barriers that participants felt were the most important *Support* barriers were:

1. Having no-one to contact for advice/clarification/help when confused by or misunderstanding the presented material (nine votes).
2. When the course is finished you get fed up doing it on your own (seven votes).
3. Lack of human contact (six votes).

#### Technological barriers

With regards to *Technological* barriers, participants highlighted the difficulty navigating eHealth programs, uploading information, opening links to program materials and the lack of user-friendly interfaces as barriers to the use of eHealth technology. Participants also identified specific technological functions of the eHealth programs as barriers, such as difficulty with viewing videos on small monitors, starting/pausing/stopping videos and changing text to a larger font for easier reading. Participants again felt that their needs as older adults were not being met and felt that this type of technology feels like it is centred around younger populations and not made with their age group in mind.

The three barriers that participants felt were the most important *Technological* barriers were:

1. Problems with passwords (five votes).
2. Difficulty navigating programmes (four votes).
  Lack of user-friendly interface (four votes).
  Difficulty setting up Wii-like interactive devices (four votes)
3. Difficulty changing text to a larger font for easier reading (two votes).
  Failure to understand how to open links online (two votes).
  Difficulty uploading certain information (two votes).

#### Personal barriers

In the next category focused on *Personal* barriers*,* participants highlighted that mastering technology can be intimidating for middle-aged and older adults which can act as a barrier to eHealth technology use, with many lacking the confidence and ability needed to try new things. Personal difficulty with keeping up with the momentum required by eHealth programs and the failure to establish and adhere to a routine while using eHealth programs were also identified as barriers. Participants highlighted the importance of retaining control over their personal information and that the nervousness and lack of trust that can accompany allowing others to use or share this information can also act as a barrier. The effect that medication can have on participants and their ability to partake and complete these programs was also noted by some participants.

The three barriers that participants felt were the most important *Personal* barriers were:

1. Feeling nervous about personal data or name being used or shared (10 votes).
2. Mastering technology can be intimidating (seven votes).
3. Difficulty with keeping up momentum (five votes).

#### Computer literacy barriers

In relation to *Computer Literacy*, participants felt that there was a presumption on the part of eHealth technology designers that everybody has the keyboard and computer skills necessary to successfully partake in and complete eHealth programs. Participants highlighted that older adults may not be familiar with computers, have much experience using computers or understand technology in general, all of which can act as barriers to eHealth technology use. It was also highlighted that even if older adults do have experience with or an understanding of computers, solving computer problems can be difficult and may reduce a person’s ability to complete an eHealth technology course.

The three barriers that participants felt were the most important *Computer Literacy* barriers were:

1. Solving computer problems may be difficult (10 votes).
2. Lack of familiarity with/knowledge of technology (nine votes).
3. Older people may not have experience with computers (four votes).

#### Accessibility barriers

Finally, barriers related to *Accessibility* were considered. Participants identified that not all older adults have access to the internet. It was also noted that there is poor-quality internet available in some parts of the country, specifically some rural areas which may act as a barrier to using this technology. The cost of accessing the facilities needed to use eHealth technology was also identified by participants as a barrier to using eHealth technology.

The three barriers that participants felt were the most important *Accessibility* barriers were:

1. Absence of WIFI in local area (nine votes).
2. Rural areas not having internet connections (seven votes).
3. Not all older adults having access to the internet (seven votes).

#### User Needs

**Figure 3.**
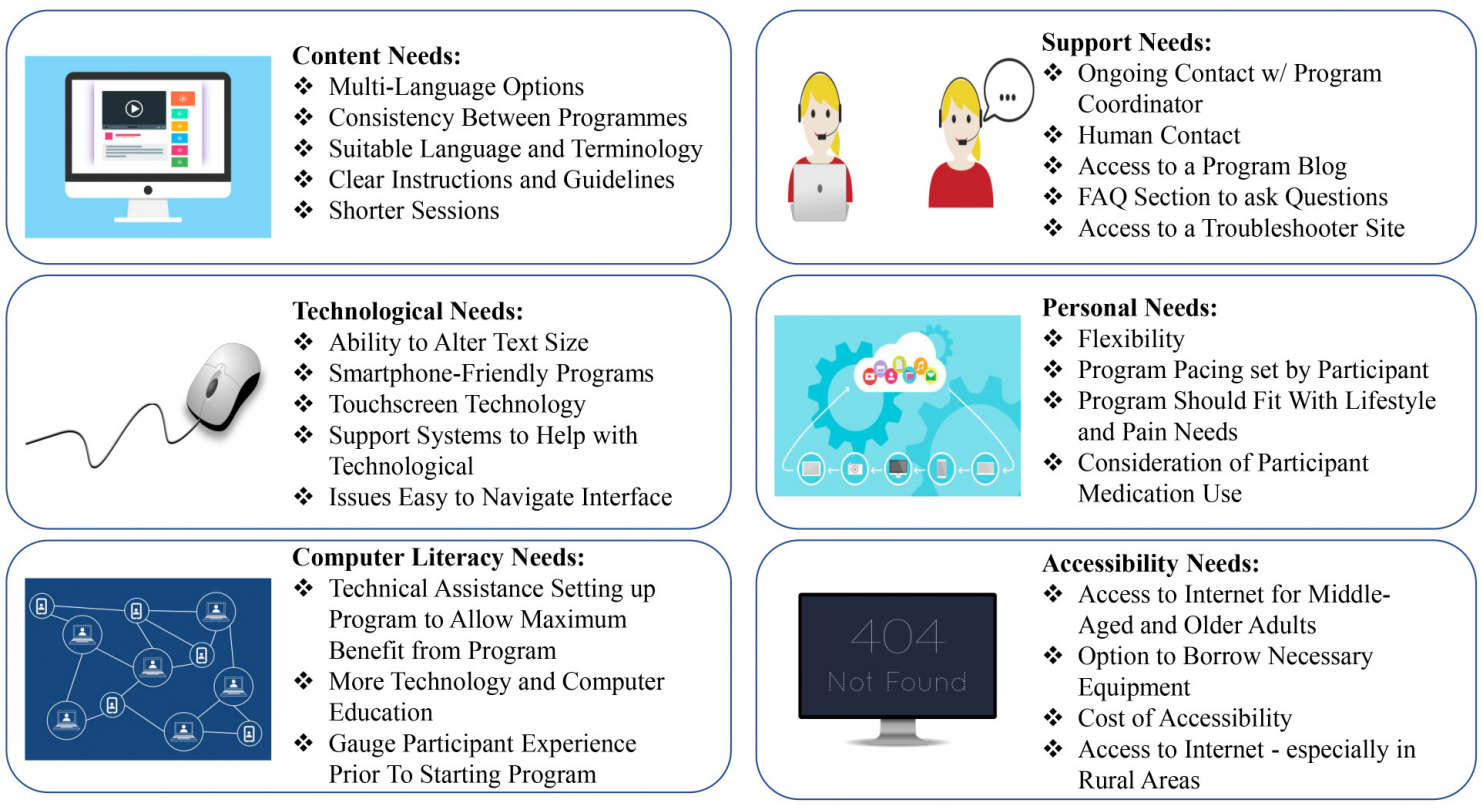
Overview of needs generated in each category.

**Figure 4.**
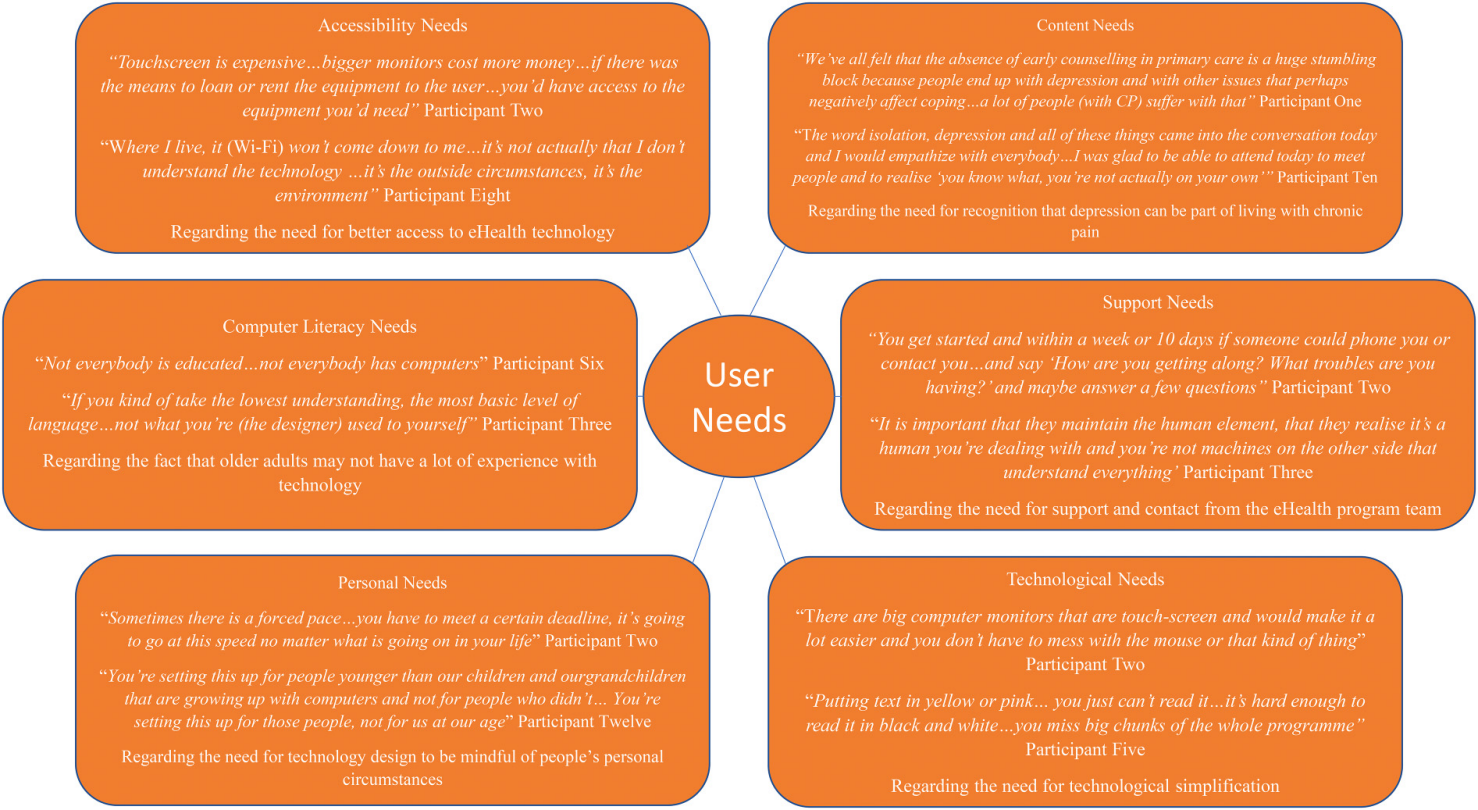
Sample of participants’ quotes for each user needs category.

Building upon the generation of barriers in the previous section, workshop participants engaged in an SBD task to identify what middle-aged and older adults need within eHealth programs to assist them with the self-management of their CP. In response to the scenarios (see Appendix B), participants generated a wide range of user needs and requirements for CP self-management eHealth technology (see Appendix C). These needs were divided into the pre-existing six categories that were used for categorising the barriers: Content Needs, Support Needs, Technological Needs, Personal Needs, Computer Literacy Needs, and Accessibility Needs. Figure 3 presents an overview of the needs generated in each category. Figure 4 provides a selection of participant’s quotes in relation to each category.

#### Content needs

The Content Needs category contains the largest number of generated needs. This category includes a variety of needs relating to the content within eHealth programs which participants felt would be beneficial for aiding older adults with the self-management of their CP. The *Content* barrier that participants felt was most important related to the absence of acknowledgement that counselling in primary care can be of some benefit to people suffering from CP. In response, participants noted that as users of eHealth technology they required more information about counselling in primary care so that they could benefit from counselling to help them cope with their CP.

The second most important Content Barrier was relating to the lack of recognition within the content of eHealth programs that depression can be a part of suffering from CP. Participants responded to this barrier by highlighting the need for relevant information so older adults can educate themselves on the latest methodologies and techniques for managing their pain issues.

The barrier that participants voted the third most important in the *Content* category was related to the lack of adequate information to assist middle-aged and older adults in the self-management of their CP. Participants responded by outlining needs that involved their age and CP situation being taken into consideration when creating eHealth programs and content that facilitates habit formation so that middle-aged and older adults can learn exercises and other techniques that will aid with the self-management of their CP at home. Participants also highlighted the need for content that would support them communicating the seriousness of their CP to family and loved ones. The need for access to online content that can inform middle-aged and older adults of the latest techniques and methodologies for CP self-management was also expressed, with some participants desiring more information about what they are entitled to within the health service that could aid with the self-management of their CP.

#### Support needs

The category of support needs highlights the feelings of impersonality that computers and using eHealth computer programs can foster and the desire that older adults have for contact with and support and help from other people. The Support barrier that participants felt was most important to them as middle-aged and older adults with CP was not having anybody to contact for advice, clarification or help if they were confused by or were unable to understand the program content. Participants responded by identifying the need for ongoing contact from a member of the program team via chat, phone, video conference or email in order to receive help with technical problems or get clarification on terminology. Others suggested a blog or a place where participants could post questions/problems and receive answers from the programme coordinator.

Participants also felt frustrated by having done previous courses without assistance from or interaction with other people. The need for on-going contact with a member of the team for encouragement and help staying involved with the program was highlighted by Participants in response to this.

The third most important support barrier that participants identified was the lack of human contact that is involved in eHealth programs. User needs relating to interaction with other participants in order to share experiences and learn from others were mentioned by several participants. This interaction was proposed both online in program-specific forums or chatrooms and in personal interactions such as support groups or social meet-ups.

#### Technological needs

The category of technological needs includes needs relating to difficulties that older adults have navigating eHealth programs, their desire for the simplification of these programs by, for example, incorporating touchscreen technology, and the option to borrow the equipment necessary to gain optimum satisfaction from eHealth programs. The *Technological* barrier that participants felt was most important were the issues that can sometimes arise with passwords. The response to this barrier included needs that related to the availability of support systems such as program coordinator feedback, the ability to ask questions about problems that participants encounter and the inclusion of a Frequently Asked Questions (FAQ) section that is updated as queries and problems arise for programme users.

An example of one of the technological barriers that participants ranked second most important related to the difficulties that can sometimes arise in setting up more complicated Wii-like devices for the purposes of using eHealth programs. Participants responded to this barrier with suggestions for the introduction of smartphone-friendly eHealth programs, technology which older adults may have more access to than, for example, a laptop with certain requirements or specifications. It was also suggested that future eHealth programmes utilise touchscreen technology.

One technological barrier that participants ranked as the third most important was to do with the difficulties that can arise trying to make the size of the onscreen writing bigger and easier to read. Participants responded saying that they need easy-to-use controls for font type, text size and screen brightness, which would allow for the comfortable control of what is being viewed.

#### Personal needs

The personal needs category represents the needs that participants have relating to their own personal circumstances. The first barrier that participants felt was most important were feelings of nervousness and untrustworthiness in relation to allowing others to use or share their personal information. Participants replied to this barrier by identifying the need for a secure program and website that is impenetrable to spam and other harmful intrusions.

The second personal barrier that participants felt was important was that mastering technology can be intimidating for older adults. Participants identified the need for the language used in eHealth programs to be made simple for the middle-aged and older adults who have not been born into a technological world.

Participants also felt that the pacing of eHealth programs and the difficulty that middle-aged and older adults can have keeping up with the momentum of the programme can act as a barrier. Participants identified the need for programme flexibility in relation to when they need to complete program modules and the fact that conditions such as CP can impede people from carrying out tasks and interrupt their daily life so having the flexibility to complete program modules at a time that suits the user is very important.

#### Computer literacy needs

Within the category of computer literacy needs, participants identified the need for the acknowledgement that older adults may not have a lot of experience with technology or computers. The need for a pre-program technological ability check was also identified to ensure that people using eHealth programs have sufficient capabilities to use computers. The barrier that participants felt was most important related to the fact that solving computer problems can be difficult for older adults. Participants responded to this by highlighting the need for a house-call from technical staff to help them to set-up the program and assure that they are comfortable with it to aid progression through the program.

The second barrier in this category that participants felt was important was the lack of familiarity with and knowledge of technology that middle-aged and older adults may have. Participants again identified the need for the simplest level of language and the simplest level of technology to be used in eHealth programmes and the importance of remembering that participants will not be familiar with the scientific terminology or language that the people setting up the programme are.

Participants also highlighted that older adult’s lack of experience with computers can act as a barrier to eHealth technology use. A need that was highlighted in response to this was a way to gauge the level of experience that participants have before they partake in an eHealth program. In relation to Christopher, a character in one of the scenarios whose lack of computer experience hinders his ability to engage with an eHealth program, participants highlighted that the program facilitator should request feedback from him in terms of where his level of computer skills are at, to gauge what level he is at.

#### Accessibility needs

The accessibility needs category represents participants’ needs for better access to eHealth technology. The accessibility barriers that participants felt were most important were in relation to the absence of WIFI in the local/rural area and the lack of access to the internet that some middle-aged and older adults may have. Participants responded to this by identifying needs in relation to improved and more reliable internet connections.

Participants also highlighted that not all middle-aged and older adults have access to the internet. Participants replied to this by identifying the need for this population to have the option to borrow the equipment necessary for them to do a program.

## Discussion

For too long middle-aged and older adults with pain have not been involved in the earliest stages of the design process of solutions for their needs. The current study provides a unique contribution to the digital health literature by using CI methods in collaboration with middle-aged and older adults to identify barriers to the use of eHealth technology for the purposes of CP self-management, along with specific user needs to be designed to overcome these barriers. Participants with representative pain profiles identified 78 barriers to eHealth technology use, which were categorised into six barrier categories: (1) Content, (2) Support, (3) Technological, (4) Personal, (5) Computer Literacy, and (6) Accessibility. As a result of SBD group work, participants then generated a set of eHealth technology user needs that they, as the end users of these technologies, felt were important for inclusion in the design process to ensure optimal engagement and impact for middle-aged and older adults living with CP.

The findings of the current study are consistent with that of previous research which has investigated the needs of older adults in relation to eHealth technology use. For example, previous research has identified the need for technology to be easily accessible and user-friendly to facilitate its adoption and continued use.^55^ Indeed, participants in the current study highlighted the need for guidelines, instructions and program content to be communicated through simple language, with easily understandable information and clear instructions. The consideration of the age and health context of middle-aged and older adults with CP during the design process was also highlighted as important to enhance the usability of this technology. Participants proposed that eHealth programs provide easy playback facilities, the ability to alter the text size and screen brightness, and greater clarity and legibility of text and visual aids. eHealth programs should also utilise touchscreen technology and be made smartphone-compatible to minimise technological ability requirements on the part of middle-aged and older adults and utilise devices that are already prevalent and that this population may already be familiar with.

Accessibility needs were expressed by participants in their desire for increased countrywide access to WIFI and the ability to borrow the technology or equipment necessary if they do not have access to the required technology themselves. Where possible participants would like facilities to be put in place to borrow the necessary technology if they do not have access to it themselves.

Participants in the current study emphasised the needs of middle-aged and older adults for support and engagement throughout the process of their technology use, a finding which is consistent with previous research.^55–57^ Participants highlighted the need for ongoing contact with program staff or an assigned coach to assist with user queries, problem solving and program progression, and as a way to receive encouragement and reduce feelings of isolation. The need for support and engagement through the use of online facilities such as chatrooms forums, blogs or troubleshooting/Frequently Asked Questions websites were also highlighted. These facilities would provide a place where participants can ask questions or look for specific answers to queries or difficulties. Facilities should also be made available to enable participants to interact with other participants either online or in-person to share their experiences, combat isolation and communicate with people who understand what they are going through. eHealth programs should also contain information relevant to the broader spectrum of issues that middle-aged and older people with CP often deal with, for example, depression and anxiety and provide information relating to the availability of services, for example, counselling availability or pain clinic locations.

Previous research has found that older adults have a desire to receive training in the use of technology or devices^56^ and that their acceptance of technology may depend upon their level of knowledge of what the technology can or can’t do.^58^ Participants in the current study expressed similar needs highlighting the value of an induction session for new technology or hands-on assistance with the setting up and use of new technology to ease progression through eHealth programs without encountering technological difficulty or confusion. Participants also highlighted the need to assess participants’ technological competency so that program staff can decide if they have the ability necessary for the program or if they require assistance prior to beginning the program.

The importance of personalisation and adaptation of technology into the lives of older adults has been recommended to optimise engagement with technology.^57,58^ Similarly, participants in the current study highlighted the need for shorter eHealth program modules so participants are not inundated with too much information; greater program flexibility that allows participants to carry out modules at times that suit them; and greater control over the pacing of eHealth programs so participants are not forced to maintain the program momentum but instead set a momentum that suits them. Participants also suggested that the outline of the program be made available before a participant agrees to take part so that they can judge whether the program is suitable, beneficial or if it covers topics that they have already covered in previous programs. It was also highlighted that eHealth programs should be secure so participants can be assured that their personal information and health data is being safely transferred to the programme coordinator and being securely stored.

### Future eHealth technology design for older adults with chronic pain

As the global population increases and life expectancy increases, so too does the prevalence rate of CP. People who live with CP typically have multiple comorbidities (particularly depression and anxiety), rely heavily on the healthcare system and typically attempt to self-manage their condition over time.^59^ eHealth interventions are an important tool for healthcare practitioners to aid patients with the self-management of their condition.^3,60^ The current study adds to previous research which highlights the lack of consideration of middle-aged and older adults in the designing of eHealth technologies for the self-management of CP.^32,33^ With the emergence in recent years of new digital technologies and the rapid rate at which these technologies are advancing it is vital that these technologies are designed to the most effective level for the purposes of benefiting and improving the healthcare of those in need.

### Future eHealth technology design for other populations

The next step in advancing the work of the current study is through the design and testing of an eHealth intervention for the self-management of CP for older adults that uses the current findings as guidelines for the design of this technology. For the design of eHealth technology that successfully provides optimal engagement and maximum impact, special attention needs to be paid to what participants of the current study have identified as potential barriers to the use of these types of technology and to the needs that have been identified as important facilitators to the use of eHealth technology.

Future research could also advance the current study by using the CI methodology to identify the barriers and user needs of eHealth technology for other chronic health conditions. One example of this is the self-management of type-2 diabetes mellitus (T2DM), a chronic health condition that affects 87–91% of the 415 million people diagnosed with diabetes worldwide, and the prevalence of which is increasing rapidly.^61^ The self-management of T2DM is an important part of living with the condition and patients are recommended to engage in such self-care behaviours as taking medication, following a diet and blood sugar monitoring.^62^ Recent research investigating eHealth technology use and diabetes self-management found that participants felt frustration with the difficulties of the technology, perceived the content as irrelevant and felt frustration with the lack of face-to-face contact,^57^ making the identification of barriers and user needs for this population an important next step in the design of enhanced eHealth services.

## Conclusion

The current study provides a unique contribution to the digital health literature by using a novel qualitative methodology to incorporate the perspectives of middle-aged and older adults with CP in the earliest phase of the eHealth pain self-management technology design process through the identification of barriers to use and generating a broad set of user needs to overcome these barriers. When designing eHealth interventions, the inclusion of end users in the design stage is critical for providing researchers with a unique and valuable insight into the investigations of health conditions.^63^ The current study, through the use of the CI methodology, was able to directly involve and engage users in identifying these barriers and user needs. Through a carefully designed and facilitated process, the user group identified and discussed the specific information, affordances, and supports, which they believe are necessary to improve the design of eHealth technology. This type of participatory design process, it is hoped, will in turn provide benefits to user groups, from the improvements to eHealth technology that they have assisted in making.

## Supplemental Material

sj-docx-1-dhj-10.1177_20552076221105484 - Supplemental material for Chronic pain self-management in middle-aged and older adults: A collective intelligence approach to identifying barriers and user needs in eHealth interventions Click here for additional data file. Supplemental material, sj-docx-1-dhj-10.1177_20552076221105484 for Chronic pain self-management in middle-aged and older adults: A collective intelligence approach to identifying barriers and user needs in eHealth interventions by Paul M O’Reilly, Owen M Harney, Michael J Hogan, Caroline Mitchell, Brian E McGuire and Brian Slattery in Digital Health
