# Supplementary material for: Chronic pain self-management in middle-aged and older adults: A collective intelligence approach to identifying barriers and user needs in eHealth interventions
Source: Digit Health. 2022 Jun 7;8:20552076221105484. doi: 10.1177/20552076221105484 (PMC9185015; doi:10.1177/20552076221105484)
Supplement: sj-docx-1-dhj-10.1177_20552076221105484 - Supplemental material for Chronic pain self-management in middle-aged and older adults: A collective intelligence approach to identifying barriers and user needs in eHealth interventions [file sj-docx-1-dhj-10.1177_20552076221105484.docx]

Appendix A

Full Set of Categorised Barriers

**Table 1.** Barrier: Content

| Having difficulty following and understanding links and instructions. |
| --- |
| Failure to understand the interface. |
| Lack of information for older people to help them cope with their chronic pain on a daily basis. |
| Lack of guidelines or information to help with use. |
| Inadequate information to assist older adults self-manage their chronic pain. |
| Condescending tone of language and instructions. |
| Time allowed for the duration of the course is insufficient. |
| Lack of flow and logic to the content. |
| Multiple choice answers can often be too similar. |
| Great deal of repetition within the content. |
| Lack of information is a source of frustration. |
| Absence of advice on how to deal with questions regarding why a person can no longer participate in certain activities despite appearing perfectly well. |
| Lack of information about possible new treatments, therapies and medication for chronic pain. |
| Failure to acknowledge that depression can be part of chronic pain. |
| Absence of acknowledgment that counselling in primary care can be of some benefit to chronic pain patients. |
| Confusing amount of information i.e what is relevant to me specifically. |
| How to see course overview to see what you’re getting into – how long will it take? What time of day is it offered? |
| Failure to provide progression e.g. if already using strategies then skip to next or new levels. |
| Feedback to inform course-providers as to what is effective or not – How to measure success/failure and how to communicate that? |
| Absence of feedback that is not simply generic to everyone. |
| Stopped due to overload of information, lots of worse-case scenario. Getting definitions led to more and more sites thus hours spent checking things out and causing dread. |
| Can be time consuming following other links from main page. |
| Absence of video. This would be preference than reading old powerpoint presentations. |

**Table 2.** Barriers: Support

| Lack of human contact. |
| --- |
| Having no-one to contact for advice/clarification/help when confused by or misunderstanding the presented material. |
| Inadequate time to talk. |
| Absence of someone to talk face to face with. |
| Personal interaction versus impersonality of computers. |
| Sense of abandonment once the course was finished. |
| When the course is finished you get fed up doing it on your own. |
| Lack of contact information. |
| Lack of empathy – feel like a statistic. |
| Lack of online interactive coach via text chat or video conference link. |
| Good communication with your doctor so they know what you are going through. |

**Table 3.** Barriers: Technological

| Difficulty navigating programmes. |
| --- |
| Lack of user-friendly interface. |
| Difficulty viewing videos on small monitors. |
| Difficulty changing text to larger font for easier reading. |
| Lack of magnification options. |
| Failure to understand how to open links online. |
| Difficulty in starting/pausing/stopping videos or any type of playback. |
| Problems with passwords. |
| Difficulty uploading certain information. |
| Technology centred around younger people. |
| Difficulty in setting up Wii-like interactive devices. |
| Inability to open entire course in one document. |
| Text coloured-in makes it very difficult to read. |
| General lack of understanding of the public’s capabilities with regard to IT. |
| Absence of relevant training. |

**Table 4.** Barriers: Personal

| Mastering technology can be intimidating. |
| --- |
| Age poses a barrier and can sometimes cause embarrassment. |
| Inability to try/trust in something new. |
| Lack of confidence. |
| Feeling nervous about personal data or name being used or shared. |
| Failure to establish/adhere to a routine. |
| Difficulty with keeping up momentum. |
| Difficulty with thinking too much about the outcome. |
| Failure to make real improvement. |
| Difficulty with head and eyesight when using computer. |
| Inability to focus due to strong medication. |
| Listing medications prior to answering questions. |
| If you can’t read or write. |
| Absence of understanding of chronic pain across the health service. |
| Difficulty coping with everyday life. |
| Lack of confidence using some technology. |
| Lack of trust about whether my details are kept in confidence and secure. |
| Absence of understanding of the long-term effects of pain on the patient and partner. |

**Table 5.** Barriers: Computer Literacy

| Presumption that older adults have competent keyboard/computer skills. |
| --- |
| Older people may not have experience with computers. |
| Lack of familiarity with/knowledge of technology. |
| Lack of understanding of how to use computers. |
| Inability to understand technology. |
| Solving computer problems may be difficult. |

**Table 6.** Barriers: Accessibility

| **Barrier: Accessibility** |
| --- |
| Not all older adults have access to the internet |
| Absence of WIFI in the local area |
| Rural areas not having internet connections |
| Cost of accessibility. |
| Inadequate broadband. |

Appendix B

Scenarios

1. Mark is a 57-year-old lorry driver who was diagnosed with osteoarthritis in his left knee 1 years ago. His pain comes and goes and ranges from very mild to severe. Despite this, he manages to carry out his work duties. Since his diagnosis his GP has tried several different types of medication but these have only reduced the pain by a small amount. 6 weeks ago Mark’s GP referred him to a Pain Specialist who suggested that enrolling in an internet-based pain self-management course might help him with his pain. The specialist emailed Mark the address of the website after the consultation and he signed up to take part in the course. At a specified time each week Mark is required to watch a 50 minute video with tips on how to self-manage his pain. He is also required to complete exercises each day as part of the programme. For the last two weeks Mark has missed the video due to family commitments. He has also had to skip a few of the daily exercises due to his pain being very bad. He has asked the people who run the program if he can catch up on the sessions that he has missed but they told him that once a new session is released you cannot go back and do older sessions. He is considering dropping out of the programme as it is not fitting in with his day-to-day life. He knows that he may miss out on an opportunity to reduce his pain and improve his daily life but he feels that he is unable to keep up with the demands of the programme. Mark feels he needs a programme which provides some more flexibility which will be easier to work with on a day-to-day basis.

2. Claire is a 74-year-old woman who has suffered from chronic lower back pain for the last 10 years. After medication and nerve blocking procedures were unsuccessful in reducing her pain, her Pain Specialist suggested that she try an online chronic pain management programme, which consists of methods to manage chronic pain including videos of people talking about how they self-manage their chronic pain. Although she was told that this programme has been shown to be very effective for reducing pain, Claire stopped using the programme after 2 or 3 attempts because she found the phrases and language that were used throughout the programme very complicated and difficult to understand. Claire also had difficulty navigating the programme and found it hard to relate to the videos due to the people in the videos being very young. She spoke to her Pain Specialist about the problems that she was having and he suggested that she email the people who run the programme. Claire feels she needs access to a programme which is more accessible in terms of the language used, and more user-friendly, but this is not what is currently available. She also feels that the use of people closer to her own age would make the program more relatable.

3. Christopher is a 69-year-old farmer who has suffered from chronic pain ever since having an operation on his upper back 6 years ago. He has lived alone on his farm since his wife passed away 10 years ago. On his last house-call his GP suggested that Christopher attend a Pain Specialist in the city as none of the medication he has prescribed has been effective but Christopher lives in a very isolated part of the country and finds it hard to make time to get away from his farm. Christopher eventually spoke to a Pain Specialist on the phone and she suggested that he try an online chronic pain self-management programme to help him with his pain. He was very reluctant to use the program as he has very little experience with computers and did not believe that a computer programme would be able to help him with his pain. Despite his reservations he asked a neighbour to help him use the program but he found the programmes extremely difficult to understand and found it difficult to read some of the text on the screen as it was very small. Both Christopher and the Pain Specialist feel there is a need for more technical support for patients like Christopher, and more effort made to make the programmes user-friendly and easy to understand. Without this, Christopher stopped trying to use the program.

4. Sarah has just started an online chronic pain self-management course on the recommendation of her Pain Specialist. The course consists of one session per week over 8 weeks and contains a combination of instructional videos and workbooks. Participants are asked to log on and do each session at whatever time suits them during each week. Sarah successfully completed the introductory session to the program but is finding the second session difficult to navigate and much more complicated than the introductory session. She asks her son to help her with the stopping and starting of the videos and how to move from one screen to the next as the program does not explain how to do this. However, during the next session Sarah encounters a problem, and her son is not there to help. Sarah is frustrated with the program and feels more support should be available to help patients to navigate each session, and that the instructions should be clearer.

Appendix C

User Needs

**Table 1.** User Needs: Content

| I want links to group that does an activity e.g. hydrotherapy so that I can try a variety of things at little cost. |
| --- |
| I want clear information that is easily understood so that I can understand what the programme is about. |
| I want video footage so that I feel I’m interacting with another person and it would make it easier when you can’t use your hands for long. |
| I want adequate information for my own problems so that I can understand how to manage my chronic pain. |
| I want advice on habit formation so that I can continue to do the exercises/techniques that will help me improve physical movement. |
| I want knowledge of available techniques/resources that will enable me to become more mobile. |
| I want a series of daily exercises (10-15 mins daily) that will help knee/back pain. |
| I want online advice to remain online so that I can be reminded of good habits when I get lazy & lapse. |
| I want a list of content of each session prior to signing up so that I can decide if it’s worth my energy. |
| I want to be able to revisit each session as much as I need e.g. printout/video so that I can take in the information provided – also depending on health something can help at times and not at others. |
| I want clear concise sessions stating desired outcomes so that I will know if this is beneficial to me. |
| I want a way to measure whether each session is having a positive impact so that I can see how effective each thing is so I will know where to focus my attention after the course. |
| I want biofeedback to test to what extent techniques are working so that I can see how effective each thing is so I will know where to focus my attention after the course. |
| I want content prepared with my age and situation properly accommodated for so that I can focus on learning and taking proper actions, rather than being confused or irritated or frustrated. |
| I want language and terminology designed for non-technical older adults so that I can focus on learning and taking proper actions, rather than being confused or irritated or frustrated. |
| I want limited duration videos with easy start/stop provisions so that I can maintain focus and learn in smaller ‘bites’ or chunks. |
| I want text version of videos with hypertext links so that I can skim content quickly and/or study it in detail and print it out to read anywhere. |
| I want properly spoken/written English free of verbal tics, excessive ‘likes’ so that I can focus on the content & not be irritated by speaker’s poor speech habits. |
| I want the options to have multi-language so that I can do the programme in my native language. |
| I want links to related sites that provide additional information so that I can educate myself on the latest methodologies and techniques for managing my pain issues. |
| I want access to online programmes yoga, CBT etc so that I can use these to alleviate my pain. |
| I want to be able to access resources specific to my pain so that I can try these at home to see if they benefit me. |
| I want access/contact to professionals that excel in their area of expertise so that I can spend my money wisely. |
| I want more information about what we are entitled to. |
| I want more information about counselling so that I can benefit from counselling to help me cope. |
| I want to be able to go online and find the information that I’m looking for instead of being led in a direction I don’t want to be in. |
| I want information on medication and support systems so that I can best access what is good for me. |
| I want bullet points so that I can access program at my leisure and get advice. |
| I want shorter online time so that I can move freely. |
| If all course does not suit – a synopsis. |
| I want shorter programmes so that I can feel I can continue & complete the program. |
| I want information on what to do if unable to watch each session – tips or advice on how to get the best from the programme so that I am not missing out on information. |
| I want encouragement about mobility – hydrotherapy/physiotherapy – to stop my very sedentary lifestyle. |
| I want concise data so that I can avoid going from link to link to link. |
| I want advice on daily management when faced with difficulties so that I can become more sociable & confident. |
| I want those setting the content of the programme to make sure they maintain the human element in the questions as much as possible. |
| I want a more gradual introduction to each new programme so that when moving from introduction to the next modules I am able for it. |
| I want YouTube videos during course to identify problems with content as they arrive. |
| I want consistency between programmes. |
| I want Email or hard copy of material. |
| I want a person-centred approach. |
| I want User-friendly content. |
| I want Specific tailored online resources. |
| I want more online video which would be easier to absorb. |
| I want a sequence of actions, exercises to perform to. |
| I want a content overview, introduction – what is covered, how long will it take? |
| I want online web/video from local experts/consultants in pain management. |
| I want it stated clearly how the course differs from reading or internet. |
| I want the ability to link to related materials, explanations, definitions e.g. hyperlinks. |

**Table 2.** User Needs: Support

| I want a coach assigned to me who can respond to me via chat, phone, video conference or email so that I can receive help with technical problems or get clarification on terminology or get occasional encouragement to continue. |
| --- |
| I want a blog or a place to post questions online so that I can get answers - a FAQ would be good too but keep adding to it and refining it. |
| I want ongoing contact from a program coordinator/coach to monitor or check on my progress or lack thereof and provide encouragement/assistance so that I can stay involved, overcome simple obstacles and resolve difficulties with the technology. |
| I want support to communicate my condition with family so that I can give an understanding of what my chronic pain consists of to others – what you are going through when you feel alienated from social activities. |
| I want more interaction with ‘people’ so that I can share my experience, learn from others, feel less isolated and stay in touch with other survivors who understand difficulties and tell each other things that help. |
| I want a contact person so that I can check the suitability of physical exercises. |
| I want a pain specialist to run the programme so that I can access information and help when I need it – provide links to help – ability to send questions to pain specialist online. |
| I want a list of local contacts so that I can meet up/join a support group/share problems and solutions. |
| I want a troubleshooter site available - to aid people with problems on navigation etc. |
| I want a group chat or forum so that I can get support. |
| I want a long-term group or forum to be set up to continue to meet people with chronic pain. |
| I want more access to other users on the course. |
| I want someone to talk to 24/7 to alleviate panic/anxiety online via a group chatroom or forum. |
| I want more support so that I can do this without help. |
| I want continuous feedback. |
| I want a Help/Assistant icon. |
| I want designers to allow for 1-on-1 counselling sessions to help the patient get through the program. |

**Table 3.** User Needs: Technological

| I want large easy-to-use controls of text size (and maybe font), screen brightness, black on white or white on black – so that I can increase text size, navigate with simplicity and control comfort of what is being viewed. |
| --- |
| I want the option for programmes to use touch screen so that I can have better user-friendly access and usage. |
| I want a trial run in person prior to start of course so that technical issues can be addressed. |
| I want loan of computer/laptop for those that don’t have big enough screen etc so that I can overcome technical issues – issue to entire group to standardise technology used. |
| I want text and visual aids to be clear and readable so that I can understand the content in total and make sense of the complete programme. |
| I want the ability to give continuous feedback so that I can remember content and give feedback as retention of information can be difficult. |
| I want one document to print out clearly and read so that I can refer to it without using a PC or other device. |
| I want an opportunity to watch video again so that I can catch up. |
| I want to retain site online so that I can access when needed. |
| I want each programme set so it is easy to navigate. |
| I want the ability to review, go back, stop/start or restart at any point. |
| I want it to be smartphone friendly. |
| I want there to be a support system so that I can get help with the parts that are confusing me. |

**Table 4.** User Needs: Personal

| I want flexibility to log on and off the course with no restriction of time. |
| --- |
| I want flexibility so that I can catch up if I miss a session. |
| I want the course to be flexible so that it fits in with my lifestyle and pain needs. |
| I want open morning of complimentary therapies to work in conjunction with course so that I can try things – taster sessions. |
| I want the program to be flexible to help with the pacing. |
| I want advice re: combining medication/exercise so that I can avoid over-reliance on meds. |
| I want to be able to clearly and simply communicate and explain my condition with simple sentences so that I can communicate with friends and family. |
| I want to avoid reliance on medications - Hydrotherapy/physiotherapy options. |
| I want to develop better habits with means of measurement of benefit/efficacy. |
| I want a secure site. |
| I want the computer language dumbed down. |

**Table 5.** User Needs: Computer Literacy

| I want the option to have someone to do house-calls to set up user so that I can progress self-help management and education on pain management. |
| --- |
| I want more education. |
| I want language and terminology designed for non-technical adult. |
| I want everything simpler and more accessible. |
| I want to be assessed as to whether I have the technical ability necessary to do the program before I start. |

**Table 6.** User Needs: Accessibility

| I want better wifi in rural area so that I can log on fast and stay on without the programme crashing. |
| --- |
| I want a loan/rental of equipment. |

Appendix D

User Needs Sheet

When generating ideas for the online or internet-connected technology, consider these domains:

- **Information and knowledge** (e.g. “I want clear instructions and guidelines provided throughout, so I can get the most from a CP self-management programme.”)
- **Communication support** (e.g. “ I want more supports included, so I don't feel like I am doing the program on my own.”)
- **Usability support** (e.g. “I want the option to magnify the screen and text, so that I can read the content on the screen.”)
- **Behavioural support** (e.g. “I want advice on habit formation, so that I can build my chronic pain self-management programme into my daily routine.”)

| **As a user** | **I want ……** | **, so that I can …..** |
| --- | --- | --- |
|  |  |  |
|  |  |  |
